# Ethyl pyruvate attenuated coxsackievirus B3-induced acute viral myocarditis by suppression of HMGB1/RAGE/NF-ΚB pathway

**DOI:** 10.1186/s40064-016-1857-6

**Published:** 2016-02-29

**Authors:** Ying Yu, Yong Yu, Ming Liu, Peng Yu, Guijian Liu, Yuxi Liu, Yangang Su, Hong Jiang, Ruizhen Chen

**Affiliations:** Key Laboratory of Viral Heart Diseases, Ministry of Public Health, Shanghai, 200032 China; Shanghai Institute of Cardiovascular Diseases, Zhongshan Hospital, Shanghai Medical College of Fudan University, Shanghai, 200032 China

**Keywords:** Ethyl pyruvate, Coxsackievirus B3, Myocarditis, High mobility group box 1, Receptor for advanced glycation end product, Nuclear factor-ΚB

## Abstract

Inflammation plays important roles in the pathogenesis of coxsackievirus B3 (CVB3)-induced acute viral myocarditis (AVMC). Ethyl pyruvate (EP) has been shown to be an anti-inflammatory agent. High mobility group box 1 (HMGB1)/receptor for advanced glycation end product (RAGE)/nuclear factor (NF)-ΚB pathway has close relation with inflammatory responses. Here, we investigated the effects of EP on CVB3-induced AVMC and potential mechanisms. The mice with AVMC were treated with EP (40 or 80 mg/kg/day) from day 5 to day 7 post-infection. EP significantly decreased the mortality of mice with AVMC. H&E staining and immunohistochemistry for HMGB1 demonstrated less inflammatory lesions and fewer abnormal location of HMGB1 in the hearts of AVMC mice receiving EP. Immuoblot showed that EP significantly inhibited the levels of HMGB1, RAGE, phospho(p)-NF-ΚB and p-I-ΚBα, and raised I-ΚBα expression in the hearts of AVMC mice. Furthermore, real-time PCR and Elisa displayed decreased levels of HMGB1, TNF-α, IL-1β, IL-17 and increased levels of IL-10 in the hearts and serum of AVMC mice treated with EP. Our findings suggest that EP protects against CVB3-induced AVMC that is associated with inhibition of HMGB1/RAGE/NF-ΚB pathway.

## Background

Enteroviruses such as coxsackievirus B3 (CVB3) have been demonstrated to be the common causes of acute viral myocarditis (AVMC), which is characterized by myocardial inflammation (Kindermann et al. 2012; Corsten et al. 2012). Considerable attention has focused on treatment of this disease; however, no effective anti-inflammatory therapy has been proven.

Ethyl pyruvate, which is a simple aliphatic ester of pyruvic acid, has been identified to play an important anti-inflammatory role in animal models. For example, recent evidences have demonstrated that ethyl pyruvate significantly decreased the mortality of the mice with lethal sepsis and acute lung injury (Ulloa et al. 2002; Shang et al. 2009), and ameliorated severe acute pancreatitis (Yang et al. 2009). In the animal model of liver failure, ethyl pyruvate administration was found to improve hepatic histopathology and reduce the levels of inflammatory cytokines (Woo et al. 2004). However, little is known whether ethyl pyruvate can inhibit CVB3-induced AVMC.

Substantial evidences have confirmed that the main anti-inflammatory mechanism of ethyl pyruvate is attributed to the inhibition of levels and secretion of high mobility group box 1 (HMGB1; Ulloa et al. 2002; Wang et al. 2013), which is an important pro-inflammatory cytokine in activation of inflammatory reaction. It is known that HMGB1 releases from nuclei into extracellular milieu in response to exogenous and endogenous stimuli, subsequently, it binds to receptor for advanced glycation end products (RAGE), which is a known receptor for HMGB1, and then activates nuclear factor (NF)-ΚB to mediate the production and release of other cytokines, such as IFN-γ, TNF-α, IL-1β, IL-6, etc. (Ulloa and Messmer 2006; Xu et al. 2010).

In the present study, we evaluated whether ethyl pyruvate attenuated CVB3-induced AVMC via down-regulation of HMGB1/RAGE/NF-ΚB pathway. Our results demonstrated that ethyl pyruvate significantly decreased mortality and reduced myocardial inflammation, which was closely associated with inhibition of HMGB1/RAGE/NF-ΚB pathway in a murine model of AVMC.

## Methods

### Virus

CVB3 (Nancy strain), which was maintained by passage through Hela cells, was preserved in Key Laboratory of Viral Heart Diseases, Zhongshan Hospital. As our previous studies (Chen et al. 2011; Wang et al. 2014), the virus titer was measured by median tissue culture infective dose (TCID_50_).

### Animals and animal model of AVMC

All animal protocols were approved by the Animal Care Committee of Fudan University. Male BALB/c mice (15–20 g), a susceptible mouse strain to CVB3 infection, were purchased from Shanghai SLAC Laboratory Animal Co., Ltd. According to our previous studies (Chen et al. 2011; Xie et al. 2012), mice were intraperitoneally administrated with CVB3 that was diluted in 200 μl Eagle’s minimum essential medium (EMEM; TCID_50_ = 10^7^) to establish AVMC. Sham-operated mice underwent the same procedure with intraperitoneal injection of 200 μl EMEM without CVB3.

### Ethyl pyruvate preparation

Ethyl pyruvate (Sigma-Aldrich, USA) was dissolved in a Ringer’s lactate solution (RLS) (Tianrui Pharmaceutica, China).

### Experimental protocol

In the experiment I, the mice which received CVB3 were randomly sacrificed on day 0, 1, 2, 3, 4, 5, 6, 7, 8 after the injection of CVB3 (n = 12, each group). Inflammatory lesions in the hearts were detected.

In the second experiment, sham-operated mice and AVMC mice randomly received 200 μl RLS or 200 μl ethyl pyruvate (40 or 80 mg/kg/day) from day 5 to day 7 post-infection (n = 12, each group). The mice were sacrificed on day 8 post-infection, and hearts and serum were harvested. The mortality was recorded, and inflammatory lesions were determined. Furthermore, the expression and location of HMGB1, the levels of RAGE and phospho (p)-NF-ΚB p65, NF-ΚB p65, p- IΚBα, IΚBα as well as cytokines (TNF-α, IL-1β, IL-17, IL-10) were measured.

### Hematoxylin and eosin (H&E) staining

Paraffin-embedded hearts were cut into 5-μm-thick sections and detected inflammatory lesions by HE staining, as described in previous study (Wang et al. 2014; Xie et al. 2012). Digital photographs were taken and analyzed using Image-Pro Plus 6.0 (Media Cybernetics, USA). The areas of inflammatory lesions were quantified and the extent of the lesions was expressed as the percentage of the area of positive area compared with the total area of the heart in the microscope field.

### Immunohistochemistry

All procedures were conducted according to the instruction of immunohistochemistry assay kit (Boster, China). Endogenous peroxidase activity was blocked by incubating the slides in 3 % H_2_O_2_, and non-specific binding sites were blocked with 5 % bovine serum albumin (Sigma-Aldrich, USA). Then, the sections were incubated with primary antibodies against HMGB1 (1:100, Abcam, UK) at 4 °C overnight followed by the secondary antibody provided by the kit. After that, the sections were developed in diaminobenzidine solution and counter-stained with hematoxylin.

### Western blot

Mouse heart extracts were prepared for immunoblotting as described (Xie et al. 2012). Extracts were separated by 10 % SDS-polycrylamide gel and transferred to a polyvinylidene fluorid membrane (Millipore, USA). The membranes were pre-incubated with bovine serum albumin for 1.5 h at room temperature to block endogenous immunoglobulins. The blots were then incubated with primary antibodies against HMGB1 (1:1000, Abcam, UK), RAGE (1:200, Boster, China) and phospho-NF-ΚB p65 (1:1000, Cell signaling technology, USA), NF-ΚB p65 (1:1000, Cell signaling technology, USA), phospho-IΚBα(1:1000, Cell signaling technology, USA), IΚBα (1:1000, Cell signaling technology, USA) overnight at 4 °C. Bound antibodies were detected with HRP-conjugated secondary antibody (1:8000, Jackson ImmunoResearch, USA) for 1.5 h at room temperature, followed by development using Super-signal West Pico Chemiluminescence detection system (Thermo Scientific, USA). β-actin (1:4000, KangChen Biotech, China) was used as the internal control. The levels of target molecules were analyzed by densitometry using Image Lab Software (Bio-Rad, USA). The levels of RAGE and IΚBα were expressed as ratio of the intensity of target bands to that of β-actin band, and phosphorylation of NF-ΚB p65 and IΚBα was quantified as ratio of the intensity of phospho-NF-ΚB p65 to that of NF-ΚB p65 and ratio of the intensity of phospho-IΚBα to that of IΚBα, respectively.

### RNA isolation and real-time polymerase chain reaction (PCR)

Total RNA was extracted from mouse hearts using Trizol (Invitrogen, USA) with the ratio of A260–A280 at 1.8–2.0 levels. 500 ng RNA per sample was used for reverse transcription and PCR assay (Takara, Japan). The primers used for PCR include (McClellan et al. 2015): HMGB1 sense primer: 5′-TGGCAAAGGCTGACAAGGCTC-3′, anti-sense primer: 5′-GGATGCTCGCCTTTGATTTTGG-3′; TNF-α sense primer: 5′-ACCCTCACACTCAGATCATCTT-3′, anti-sense primer: 5′-GGTTGTCTTTGAGATCCATGC-3′; IL-1β sense primer: 5′-CGCAGCAGCACATCAACAAGAGC-3′, anti-sense primer: 5′-TGTCCTCATCCTGGAAGGTCCACG-3′; IL-17 sense primer: 5′-ACCTCACACGAGGCACAAGT-3′, anti-sense primer: 5′-AGCAGCAACAGCATCAGAGAC-3′; IL-10 sense primer: 5′-GCACTACCAAAGCCACAAG-3′, anti-sense primer: 5′-CAGTAAGAGCAGGCAGCATA-3′; β-actin sense primer: 5′-GATTACTGCTCTGGCTCCTAGC-3′, anti-sense primer: 5′-GACTCATCGTACTCCTGCTTGC-3′. And procedures were carried out as follows: holding stage (denaturation) at 95 °C for 30 s, cycling stage including annealing at 95 °C for 5 s and extension at 60 °C for 34 s which was replicated 40 cycles. All reactions were conducted by duplicate for each sample and performed in Applied Biosystem^®^7500 Real-Time PCR System. The data were analyzed using comparative CT method.

### Enzyme-linked immunosorbent assay (ELISA)

According to the manufacturer’s instructions, the levels of HMGB1, TNF-α, IL-1β and IL-17 in the serum were detected using ELISA kits specific for mice (Uscn Life Science Inc, China), and serum IL-10 was assessed using a mouse IL-10 ELISA kit (R&D Systems, USA). The sensitivities for HMGB1, TNF-α, IL-1β, IL-17 and IL-10 were 22.54, 6, 2.7, 6.3 and 1.97 pg/ml, respectively. All samples were measured in triplicate.

### Statistical analysis

All analyses were carried out with SPSS16.0 statistical package (SPSS Inc, Chicago, IL). All data were shown as mean ± SEM. The comparisons of multiple groups were analyzed by one-way ANOVA with post hoc least significant difference (LSD) test. In the mortality study, time-to survival data were analyzed by the Kaplan–Meier method and compared with the log-rank test.

## Results

### The changes of inflammatory lesions in the myocardium after CVB3 infection

**Fig. 1:**
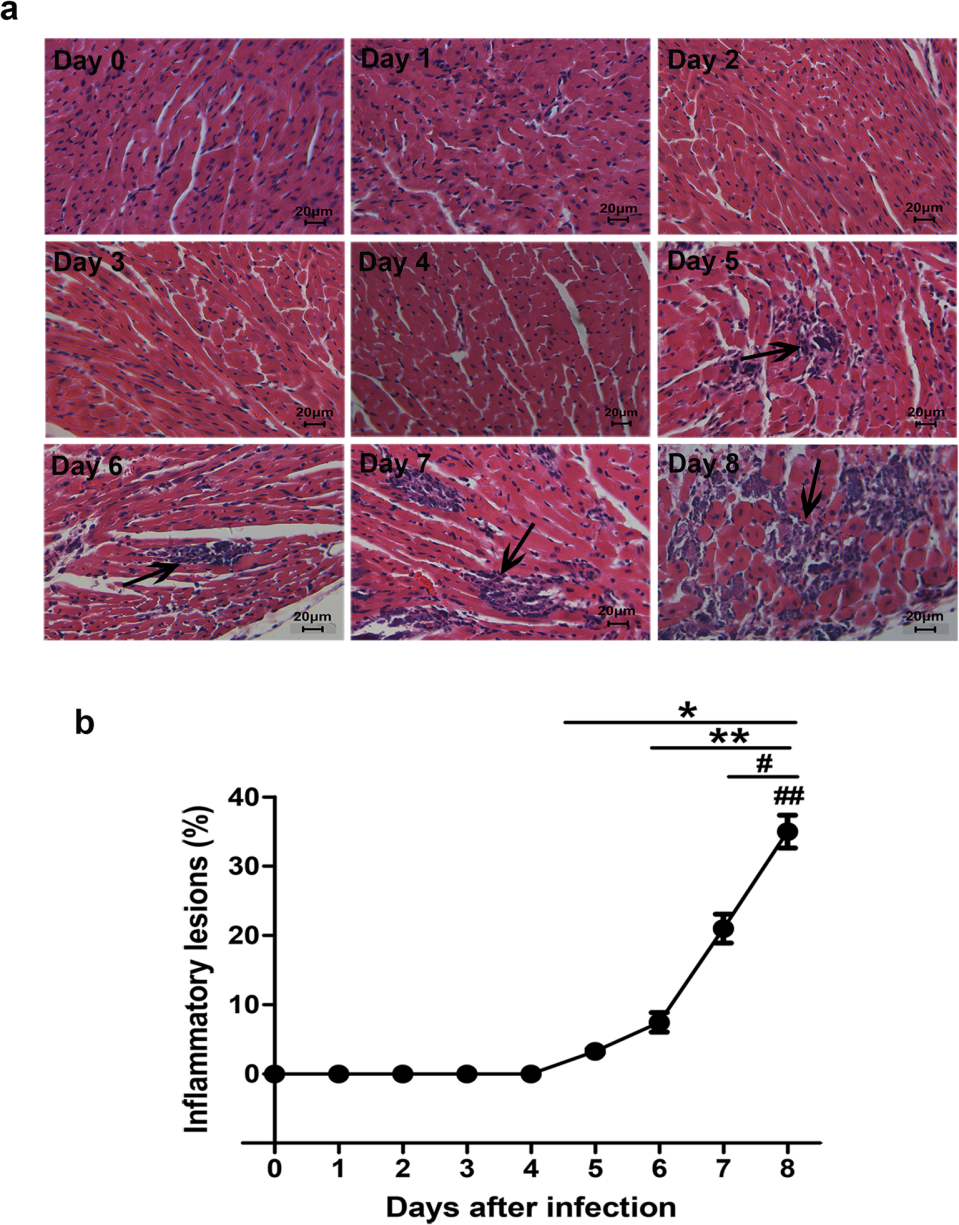
The changes of inflammatory lesions in the myocardium after CVB3 infection. The mice were sacrificed on day 0, 1, 2, 3, 4, 5, 6, 7, 8 after CVB3 injection, and the hearts were harvested. **a** A representative HE staining for inflammatory lesions in the heart tissues (×200, *arrow*). **b** Quantification of inflammatory lesions. Data were shown as mean ± SE from 6 to 12 individual experiments. *P < 0.05 versus day 0 after infection. **P < 0.05 versus day 5 after infection. ^#^P < 0.01 versus day 6 after infection. ^##^P < 0.01 versus day 7 after infection

The mice which received CVB3 were randomly sacrificed everyday within 8 days after CVB3 infection as described in methods, and inflammatory lesions in the hearts were measured. As demonstrated in previous study, inflammatory areas have been recognized as the pathological characteristic of viral myocarditis (Kindermann et al. 2012). H&E staining demonstrated inflammatory lesions were completely absent from the 1st day to the 4th day after CVB3 injection, while inflammatory areas were increasingly increased from the 5th day to the 8th day after infection (P < 0.05; Fig. 1).

### Ethyl pyruvate decreases the mortality of AVMC mice and reduces inflammatory lesions in the hearts of mice with AVMC

**Fig. 2:**
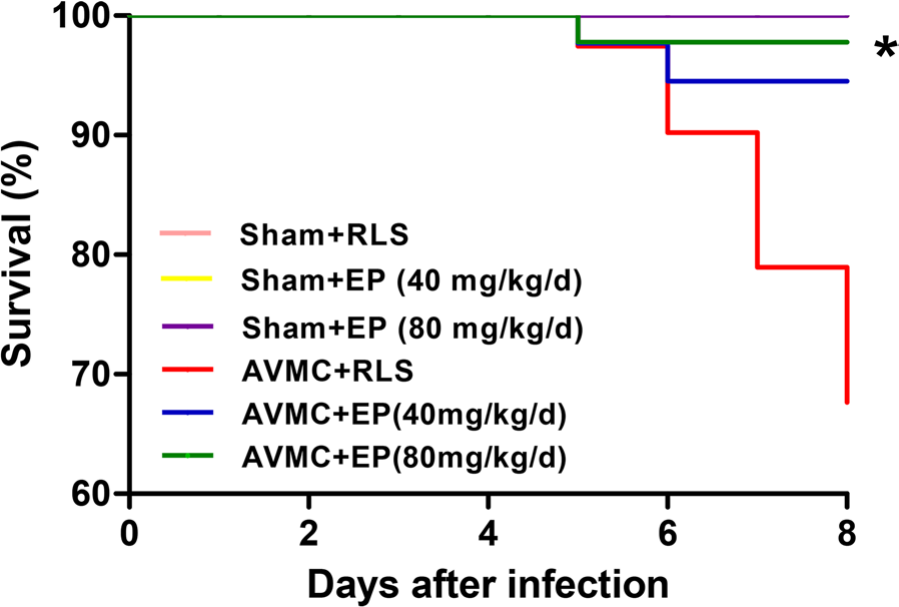
Effect of ethyl pyruvate (EP) on mortality of the mice with AVMC. The sham mice and AVMC mice were intraperitoneally administrated with Ringer’s lactate solution (RLS) or EP (40 or 80 mg/kg/day) from the 5th day to the 7th day post-infection. *P < 0.05 versus AVMC + RLS

**Fig. 3:**
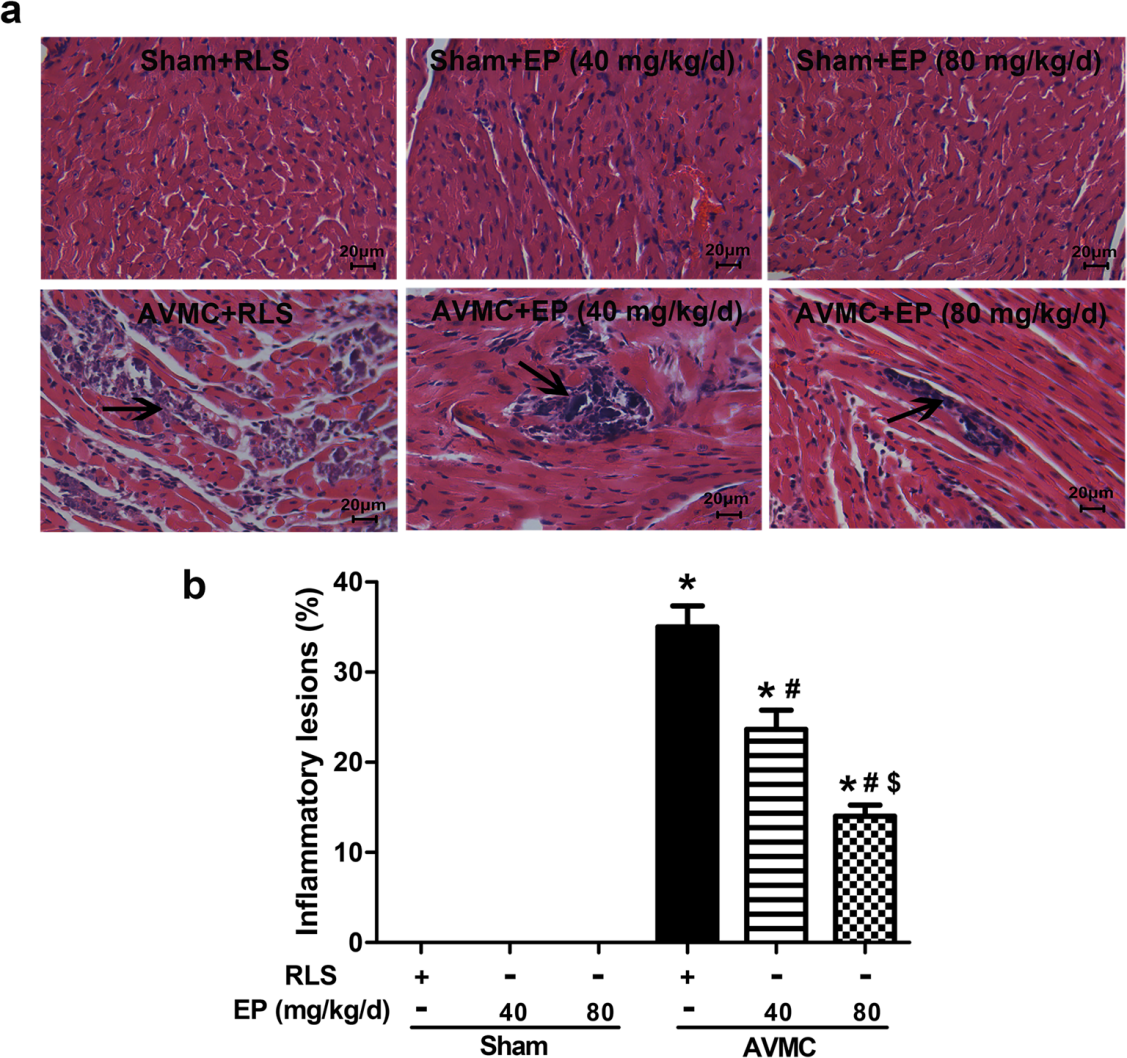
Effect of ethyl pyruvate (EP) on inflammatory lesions in heart tissues of CVB3-induced AVMC. The sham mice and AVMC mice were intraperitoneally administrated with Ringer’s lactate solution (RLS) or EP (40 or 80 mg/kg/day) from the 5th day to the 7th day post-infection. **a** A representative HE staining for inflammatory lesions in the heart tissues (×200, *arrow*). **b** Quantification of inflammatory lesions. Data were shown as mean ± SE from 6 to 12 individual experiments. *P < 0.01 versus Sham + RLS. ^#^P < 0.01 versus AVMC + RLS. ^$^P < 0.01 versus AVMC + EP (40 mg/kg/day)

To assess whether ethyl pyruvate treatment could improve AVMC, ethyl pyruvate at the dose of 40 or 80 mg/kg/day was administrated from the 5th day to the 7th day after CVB3 infection. As demonstrated in Fig. 2, the increase of mortality in AVMC mice was decreased by ethyl pyruvate in a dose-dependent manner (Fig. 2). Moreover, H&E staining demonstrated that the AVMC mice receiving ethyl pyruvate had a dramatic reduction of inflammatory lesions in the hearts, as compared with the AVMC mice treated with RLS (P < 0.01; Fig. 3). Inspiringly, high dose of ethyl pyruvate showed more effect in decreasing inflammatory lesions than low dose of ethyl pyruvate (P < 0.01; Fig. 3). These data implies that ethyl pyruvate significantly attenuates AVMC in a dose-dependent pattern.

### Ethyl pyruvate treatment inhibits the expression and release of HMGB1 in AVMC mice

**Fig. 4:**
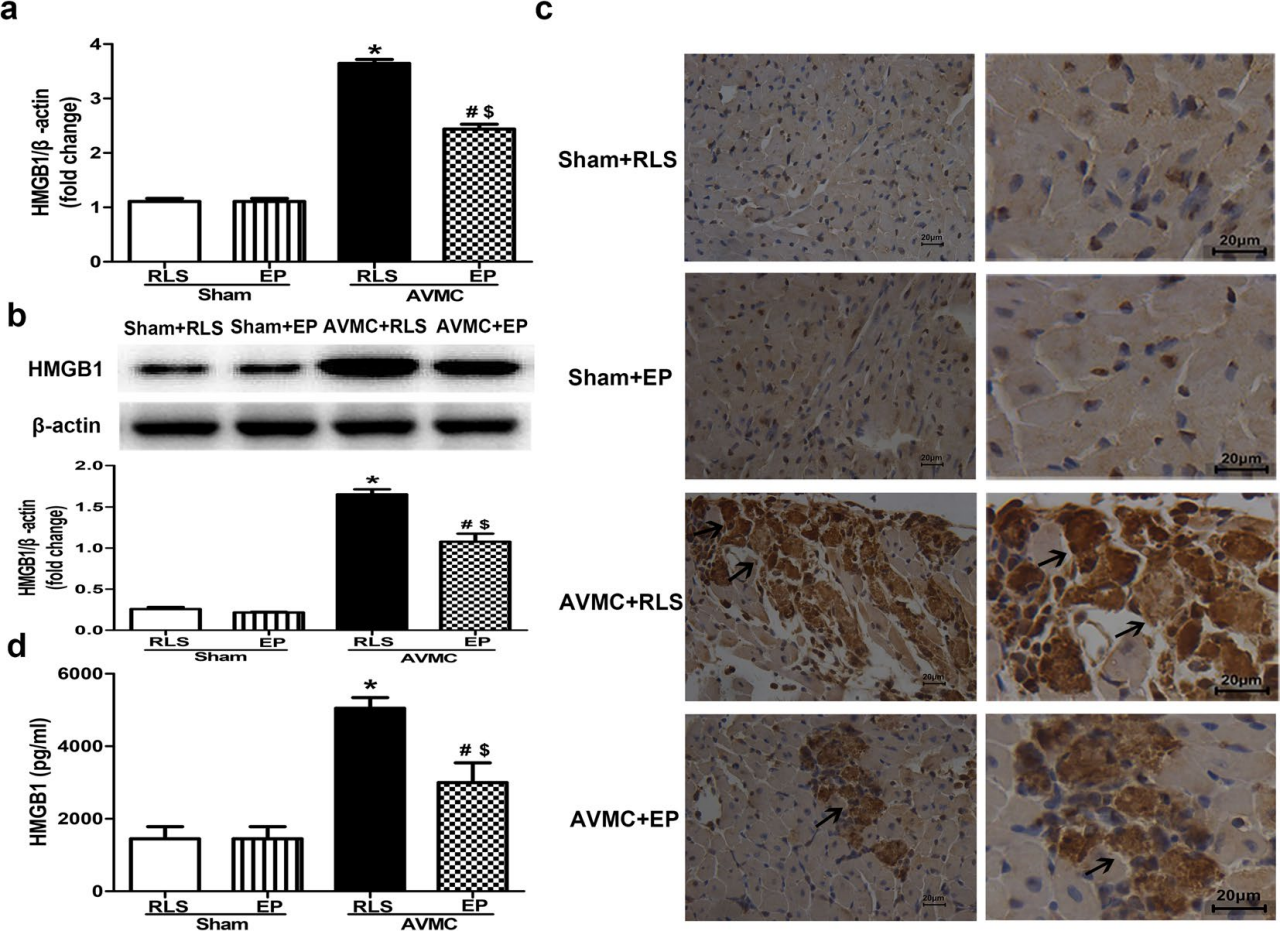
Effect of EP on the expression and release of HMGB1. The sham mice and AVMC mice were intraperitoneally administrated with Ringer’s lactate solution (RLS) or 80 mg/kg/day EP from day 5 to day 7 after CVB3 infection. **a** Real-time PCR analysis of HMGB1 in the hearts. Data were shown as mean ± SE from 6 to 12 individual experiments. *P < 0.001 versus Sham + RLS. ^#^P < 0.001 versus Sham + EP. ^$^P < 0.001 versus AVMC + RLS. **b** Immunoblot for analysis on HMGB1 expression in the hearts. The blot is showed in the *upper part*. The quantification of HMGB1 expression was shown in the *lower part*. Data were shown as mean ± SE from 6 to 12 individual experiments. *P < 0.001 versus Sham + RLS. ^#^P < 0.001 versus Sham + EP. ^$^P < 0.001 versus AVMC + RLS. **c** Immunochemistry for the localization of HMGB1 in the hearts (*Left* ×200, ×*Right* ×400). HMGB1 was predominantly located in the nuclei of the cells in the hearts of sham mice. However, in the hearts of AVMC mice, HMGB1 was not only positive in the nuclei but also positive in the cytoplasm of the cells and extracellular milieu. The *black arrows* showed abnormal location of HMGB1. **d** ELISA for quantitative analysis of serum HMGB1. Data were shown as mean ± SE from 6 to 12 individual experiments. *P < 0.001 versus Sham + RLS; ^#^ *P* < 0.05 versus Sham + EP; ^$^ *P* < 0.01 versus AVMC + RLS

Ethyl pyruvate has been recognized as a pharmacological inhibitor of HMGB1 (Ulloa et al. 2002; Shang et al. 2009; Yang et al. 2009). To investigate the effect of ethyl pyruvate on HMGB1 expression in the mice with AVMC, the expression of HMGB1 in the hearts of AVMC mice treated with 80 mg/kg/day of ethyl pyruvate was determined by real-time PCR and western blot. When compared to sham mice, HMGB1 expression was significantly elevated in the hearts of AVMC mice; however, the presence of ethyl pyruvate dramatically decreased HMGB1 levels in the hearts of mice with AVMC (P < 0.001; Fig. 4a, b).

Immunohistochemistry for HMGB1 was performed to assess whether ethyl pyruvate altered the localization of HMGB1 in the hearts of AVMC mice. As shown in Fig. 4c, HMGB1 was predominantly located in the nuclei of the cells in the hearts of sham mice, whereas HMGB1 was not only positive in the nuclei but also positive in the cytoplasm of the cells and extracellular milieu in the hearts of AVMC mice. This results suggested that CVB3 induced HMGB1 translocation from nuclei to cytoplasm and extracellular milieu in the hearts, which is consistent with the characteristics of HMGB1 release. As expected, when compared to AVMC mice treated with RLS, positive staining for HMGB1 in cytoplasm and extracellular milieu was significantly decreased in AVMC mice treated with ethyl pyruvate (Fig. 4c), indicating that ethyl pyruvate inhibited release of HMGB1 in the hearts of AVMC mice. Moreover, the levels of HMGB1 in serum were evaluated by ELISA. As shown in Fig. 4d, in comparison with sham mice, significant increase of HMGB1 was detected in the serum of AVMC mice (P < 0.001). However, serum HMGB1 in AVMC mice was reduced by ethyl pyruvate treatment (P < 0.01; Fig. 4d).

These evidences confirm that ethyl pyruvate treatment inhibits the expression and release of HMGB1 in the mice with AVMC.

### Ethyl pyruvate suppresses the expression of RAGE and activation of NF-ΚB pathway in the mice with AVMC

**Fig. 5:**
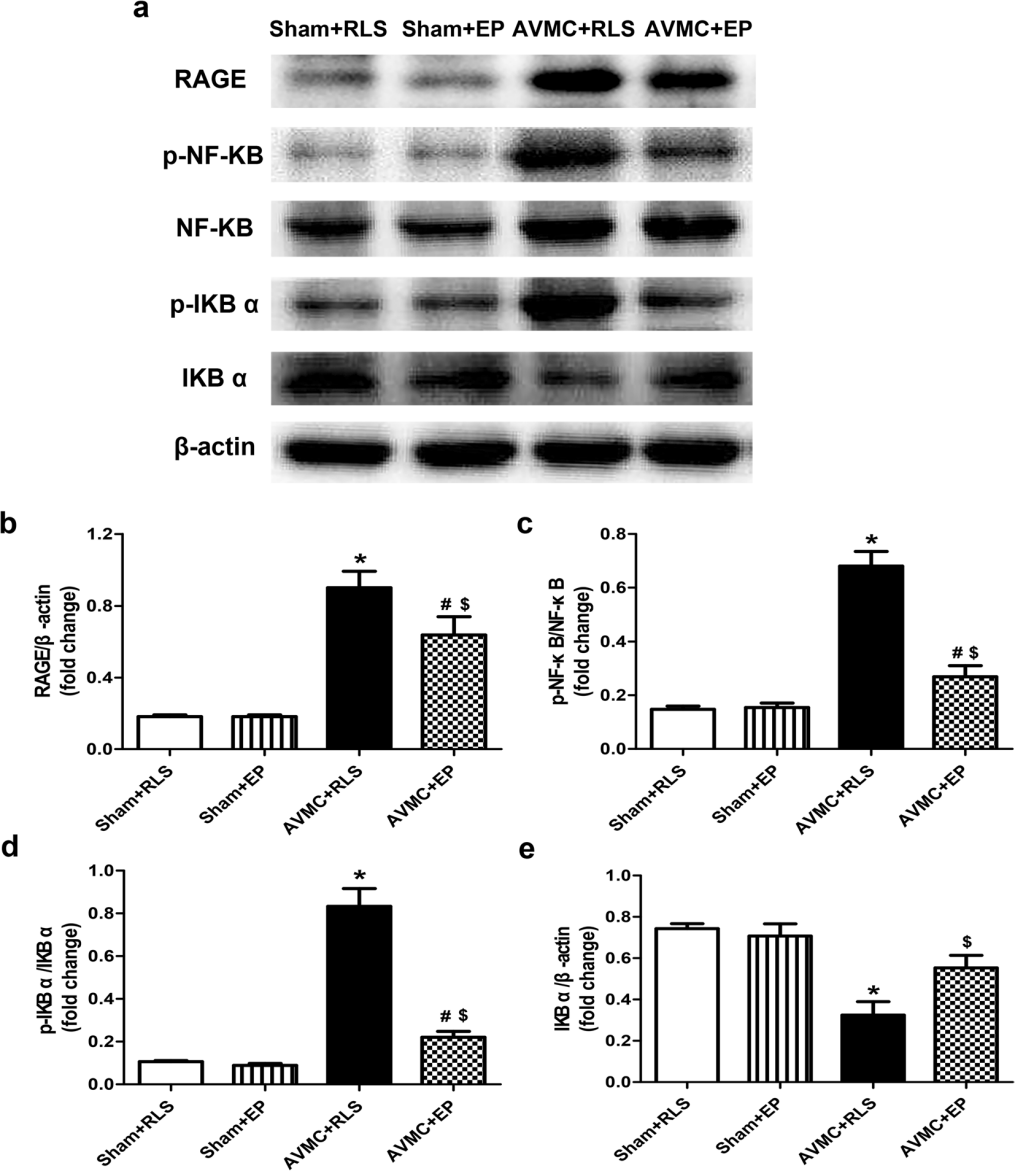
Effect of ethyl pyruvate (EP) on the expression of RAGE and NF-ΚB pathway. The sham mice and AVMC mice were intraperitoneally administrated with Ringer’s lactate solution (RLS) or 80 mg/kg/day EP from day 5 to day 7 post-infection. **a** Immunoblot for the expression of RAGE, phospho (p)-NF-ΚB p65, NF-ΚB p65, p-IΚBα and I-ΚBα. **b**–**e** Quantification of RAGE (**b**), p-NF-ΚB p65 (**c**), p-IΚBα (**d**) and I-ΚBα (**e**). Data were shown as mean ± SE from 6 to 12 individual experiments. *P < 0.01 versus Sham + RLS; ^#^ *P* < 0.05 versus Sham + EP; ^$^ *P* < 0.05 versus AVMC + RLS

It is well known that HMGB1 binds to its receptors, such as RAGE, and then activates NF-ΚB to increase the produce and release of inflammatory cytokines (Ulloa and Messmer 2006). In other word, pro-inflammatory effect of HMGB1 depends on the activation of downstream inflammatory signaling pathway. To further address the mechanism that ethyl pyruvate improved AVMC, the levels of RAGE, phospho-NF-ΚB p65, NF-ΚB p65, phospho-IΚBα and IΚBα were detected using western blot as described in methods. When compared to sham mice, RAGE expression was significantly elevated in the hearts of AVMC mice (P < 0.01). Nevertheless, ethyl pyruvate markedly decreased RAGE expression in the mice with AVMC (P < 0.05; Fig. 5a, b). Moreover, the levels of phospho-NF-ΚB p65 and phospho-IΚBα were obviously increased in AVMC mice (P < 0.01); however, the presence of ethyl pyruvate reduced phospho-NF-ΚB p65 and phospho-IΚBα (P < 0.05; Fig. 5a, c, d). In contrast with an increase in phospho-NF-ΚB p65 and phospho-IΚBα, the expression of IΚBα was substantially deceased in AVMC mice, which was recovered by ethyl pyruvate (P < 0.05; Fig. 5a, e). These results indicated that in the mice with AVMC, ethyl pyruvate treatment could reduce RAGE expression and inhibit activation of NF-ΚB by down-regulation of phosphorylation of IΚBα and suppression of degration of IΚBα.

**Fig. 6:**
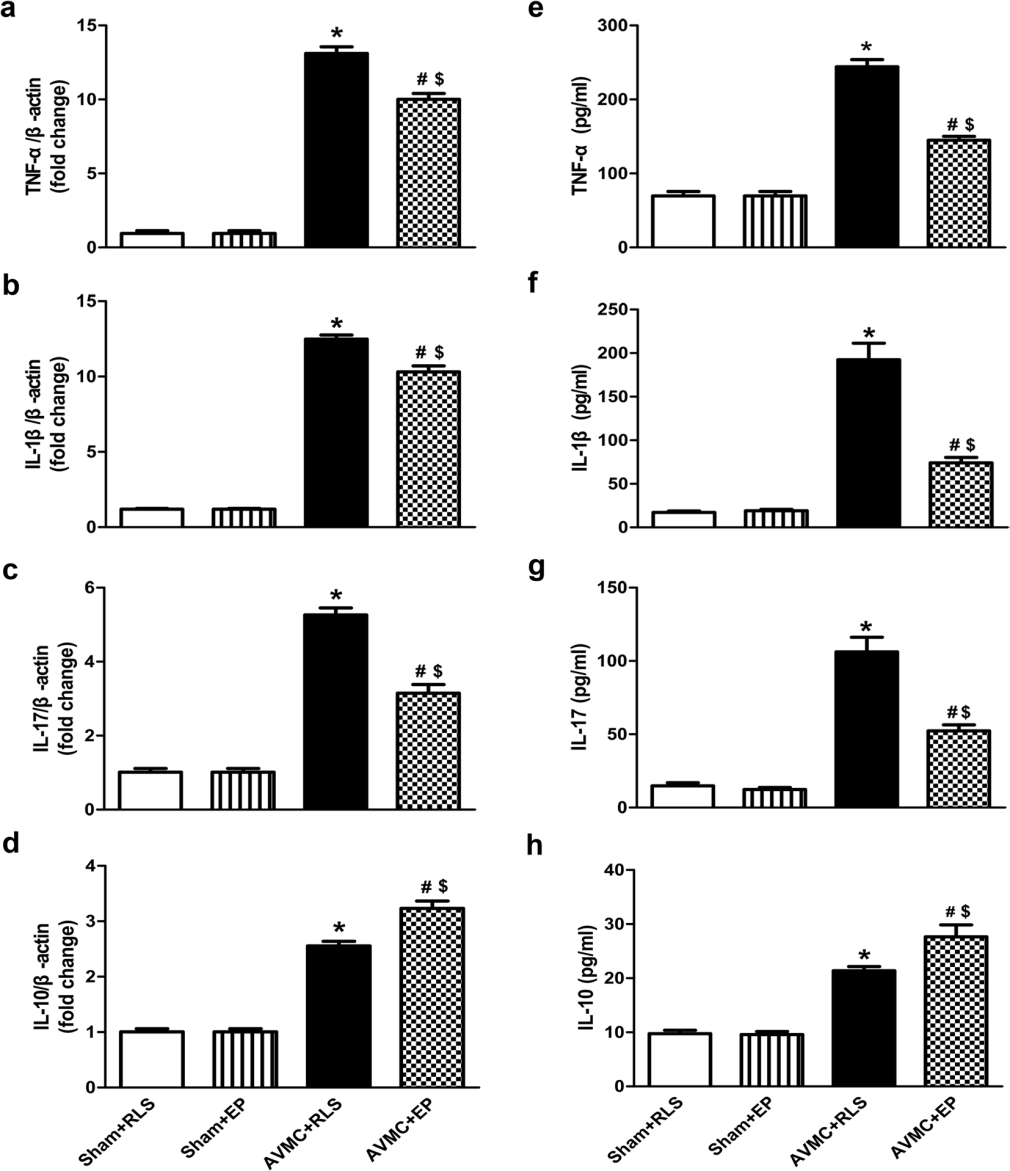
Effect of ethyl pyruvate (EP) on the cytokines. The sham mice and AVMC mice were intraperitoneally administrated with Ringer’s lactate solution (RLS) or 80 mg/kg/day EP from the 5th day to the 7th day after CVB3 infection. **a**–**d** Real-time PCR was used for analysis on the expression of TNF-α (**a**), IL-1β (**b**), IL-17 (**c**) and IL-10 (**d**) in the hearts. Data were shown as mean ± SE from 6 to 12 individual experiments. *P < 0.001 versus Sham + RLS; ^#^ *P* < 0.001 versus Sham + EP; ^$^ *P* < 0.01 versus AVMC + RLS. **e**–**h** ELISA for quantitative analysis of serum TNF-α, IL-1β, IL-17 and IL-10. Data were shown as mean ± SE from 6 to 12 individual experiments. *P < 0.001 versus Sham + RLS; ^#^ *P* < 0.001 versus Sham + EP; ^$^ *P* < 0.01 versus AVMC + RLS

Activation of NF-ΚB mediates the production and release of many cytokines, such as TNF-α, IL-1β, IL-17 and IL-10 (Xu et al. 2010; Kwok et al. 2012; Lutay et al. 2014). And previous studies have reported that these cytokines are involved in the progression of CVB3-induced acute viral myocarditis (Kindermann et al. 2012; Xie et al. 2011). Therefore, the levels of TNF-α, IL-1β, IL-17 and IL-10 were determined by real-time PCR and Elisa as described in methods. These results demonstrated that compared to sham mice, the levels of TNF-α, IL-1β, IL-17 and IL-10 were substantially elevated in the hearts and serum of AVMC mice (P < 0.001; Fig. 6). However, the presence of ethyl pyruvate significantly decreased the levels of TNF-α, IL-1β, IL-17 and increased the expression of IL-10 in the hearts and serum of AVMC mice (P < 0.01; Fig. 6), indicating that ethyl pyruvate therapy could down-regulate pro-inflammatory cytokines (TNF-α, IL-1β, IL-17) and raise anti-inflammatory cytokine (IL-10) in the hearts and serum of AVMC mice.

Together, these observations support that ethyl pyruvate reduces the expression of RAGE and suppresses activation of NF-ΚB pathway in the mice with AVMC.

## Discussion

The present study demonstrates for the first time that ethyl pyruvate treatment protects from CVB3-induced AVMC in animal model, as indicated by the facts that ethyl pyruvate significantly decreases mortality and reduces inflammatory lesions in the hearts of AVMC mice. Our findings also confirm that ethyl pyruvate treatment markedly decreases the expression and release of HMGB1, down-regulates the levels of RAGE, p-NF-ΚB p65 and p-IΚBα, and raises the expression of IΚBα in hearts of AVMC mice. In addition, ethyl pyruvate reduces the levels of pro-inflammatory cytokines (TNF-α, IL-1β, IL-17) and elevates the expression of anti-inflammatory cytokine (IL-10). These findings suggest that ethyl pyruvate may improve CVB3-induced AVMC, which may be closely associated with the reduction of HMGB1/RAGE/NF-ΚB pathway.

Ethyl pyruvate, a simple aliphatic ester derived from pyruvic acid, has been shown to be an effective anti-inflammatory agent in a variety of inflammatory diseases including severe sepsis, autoimmune hepatitis, severe acute pancreatitis (Ulloa et al. 2002; Shen et al. 2014; Luan et al. 2013). In this study, we showed that compared to the AVMC mice treated with RLS, the AVMC mice receiving ethyl pyruvate treatment from day 5 to day 7 after CVB3 injection had lower mortality and fewer inflammatory lesions in the hearts in a dose-dependent manner, suggesting that ethyl pyruvate could improve AVMC.

HMGB1, a nonhistone nuclear protein, is released from nuclei into extracellular milieu, and acts as a cytokine-like mediator in many inflammatory diseases, such as sepsis, rheumatoid arthritis (Wang et al. 1999; Shi et al. 2012). HMGB1 was up-regulated in the hearts and serum of the mice with MHCα-induced myocarditis, which was associated with increased myocardial inflammation (Su et al. 2011). In consistent with previous study, our findings also displayed elevated expression and release of HMGB1 in AVMC mice. Mounting evidences have shown that treatment with HMGB1 inhibitors such as ethyl pyruvate is beneficial in many inflammatory diseases (Musumeci et al. 2014). Ethyl pyruvate, as the first described pharmacological inhibitor of HMGB1 secretion, has the ability to specific interference with HMGB1 release from the nuclei to extracellular milieu (Ulloa and Messmer 2006). Meanwhile, ethyl pyruvate has also been reported to decrease the expression of HMGB1 (Lee et al. 2013). As demonstrated in a recent study, ethyl pyruvate treatment showed a significant decrease in HMGB1 levels that was correlated with improved lethality in the mice with sepsis (Ulloa et al. 2002). In TDI-induced asthma, ethyl pyruvate reduced the expression and release of HMGB1, which was in line with obvious improvement in neutrophil infiltration into the airway (Tang et al. 2014). Our present study revealed that treatment with ethyl pyruvate significantly reduced the expression of HMGB1 and inhibited the release of HMGB1 in the mice with AVMC, which was accompanied with decreased mortality and myocardial inflammation caused by CVB3 infection. These observations supports that ethyl pyruvate protects against AVMC via an anti-HMGB1 mechanism.

Previous studies have demonstrated that the pathogenic role of HMGB1 depends on the interaction of HMGB1 with its receptor-RAGE and the activation of downstream NF-ΚB pathway (Ulloa and Messmer 2006). The HMGB1/RAGE/NF-ΚB pathway is closely associated with inflammation in many diseases, such as systemic lupus erythematosus, diabetes, etc. (Sun et al. 2013; Kim et al. 2011). Therefore, inhibition of HMGB1-mediated inflammatory pathway has been regarded as a novel strategies for anti-HMGB1 (Kang et al. 2014). Our study showed that increase of RAGE in the hearts of AVMC mice was markedly attenuated by ethyl pyruvate treatment, which indicated the inhibition of HMGB1 pathway induced by ethyl pyruvate was via the suppression of RAGE. Recently, the results from in vivo and vitro studies indicated that ethyl pyruvate inhibited NF-ΚB activation by directly modifying p65, and thereby suppressing NF-ΚB DNA binding (Han et al. 2005). It was also reported that ethyl pyruvate decreased NF-ΚB activity by blockade of degradation of IΚBα (Shen et al. 2014). In present study, we found that administration of ethyl pyruvate could decrease the levels of phospho-IΚBα, phospho-NF-ΚB p65 and raise the expression of IΚBα in the hearts of AVMC mice, suggesting that ethyl pyruvate seemed to interfere with phosphorylation and degradation of IΚBα, and then inhibited activation of NF-ΚB. Additionally, NF-ΚB target cytokines were measured and we observed that ethyl pyruvate obviously decreased TNF-α, IL-1β and IL-17 and raised IL-10 in the hearts and serum of AVMC mice. Based on these results, we think that the main mechanism responsible for inhibition of ethyl pyruvate on CVB3-induced AVMC may be due to that it suppressed HMGB1-mediated RAGE/NF-ΚB pathway.

## Conclusion

In summary, ethyl pyruvate inhibited CVB3-induced AVMC, which is closely associated with the suppression of HMGB1/RAGE/NF-κB pathway. This study indicated that ethyl pyruvate may be a potential anti-inflammatory agent to treat AVMC.
